# New species of the genus *Ptychoptera* Meigen, 1803 (Diptera, Ptychopteridae) from Zhejiang, China with an updated key to Chinese species

**DOI:** 10.3897/zookeys.1070.67779

**Published:** 2021-11-11

**Authors:** Jiaqi Shao, Zehui Kang

**Affiliations:** 1 Key Lab of Integrated Crop Pest Management of Shandong Province, College of Plant Health and Medicine, Qingdao Agricultural University, Qingdao 266109, China Qingdao Agricultural University Qingdao China

**Keywords:** Classification, distribution, phantom crane flies, Ptychopterinae, taxonomic revision

## Abstract

We revise the taxonomy of the genus *Ptychoptera* Meigen, 1803 from Zhejiang, East China. One new species from Zhejiang, *P.tianmushana***sp. nov.**, is described and illustrated. Morphologically, the new species is most similar to *P.emeica* Kang, Xue & Zhang, 2019 and *P.formosensis* Alexander, 1924, but it can be distinguished by the coloration of the abdomen and the details of the male genitalia. In addition, *P.bellula* Alexander, 1937 is recorded from Zhejiang for the first time. Two known species from Zhejiang, *P.longwangshana* Yang & Chen, 1998 and *P.gutianshana* Yang & Chen, 1995, are redescribed and illustrated. A key to Chinese species of *Ptychoptera* is provided.

## Introduction

The genus *Ptychoptera* Meigen, 1803 is the most species-rich groups worldwide in the family Ptychopteridae, with a total number of nearly 80 known species (Rozkošný 1997; Zwick and Starý 2003; Hancock et al. 2006; Nakamura and Saigusa 2009; Ujvárosi et al. 2011; Kang et al. 2019). It is characterized by the following characters: larvae eucephalic or metapneustic, body segments with serially arranged hairs; abdominal segments 1–3 with a pair of prolegs ventrally; prolegs each with a single hook-like spine; posterior end of abdomen produced into a fairly long retractile respiratory siphon; flagellum in adults 13-segmented; wing with M_1+2_ forked; gonopod with a simple gonocoxite and a gonostylus of variable shape (Alexander 1981; Rozkošný 1997; Nakamura and Saigusa 2009).

Sixteen *Ptychoptera* species were known to occur in China, of which 10 species were published by Kang et al. (2013, 2019). Since these publications, additional new materials of the genus from Zhejiang, China have become available. Zhejiang Province is located on the southeast coast of China. As it is in the middle of subtropical zone with monsoon humid climate and superior natural conditions, Zhejiang is rich in biotic resources. One area, Mount Tianmushan is one of the biodiversity hotspots in China (Zhao et al. 2016).

**Figure 1.**
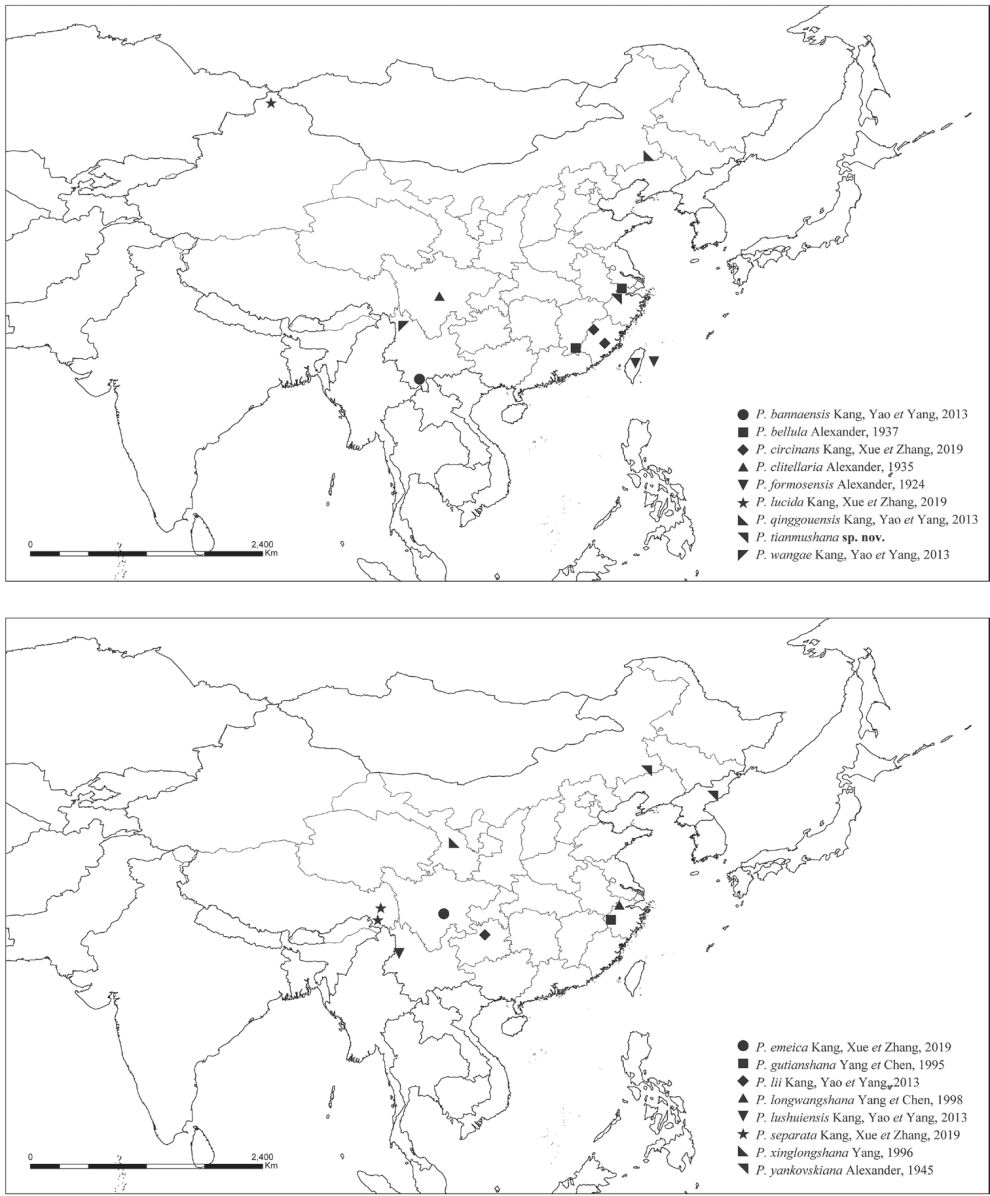
Distribution map of *Ptychoptera* from China.

Two species of *Ptychoptera* were previously recorded from Zhejiang: *P.longwangshana* Yang & Chen, 1998 and *P.gutianshana* Yang & Chen, 1995. In this paper, two *Ptychoptera* species are added to the fauna of Zhejiang, of which *P.tianmushana* sp. nov. is described and illustrated as new to science, and *P.bellula* Alexander, 1937, known previously only from Jiangxi, China, is newly recorded from Zhejiang. In addition, the two known species from Zhejiang are redescribed and illustrated based on the type specimens. A key to Chinese species of *Ptychoptera* based on the type and non-type specimens and literature is provided, and we provide an updated distribution map of *Ptychoptera* species from China (Fig. 1).

## Material and methods

Type specimen of the new species in this study was collected from Mount Tianmushan, Zhejiang, China, in June 2019 and deposited in the Entomological Museum of Qingdao Agricultural University, Shandong, China (QAU). Type and determined specimens of *P.bellula* were deposited in the National Museum of Natural History, Smithsonian Institution, Washington, DC, USA (USNM). The determined specimen of *P.bellula* was previously identified by Alexander in 1939 and the identification label is provided (Fig. 4f). Type specimens of *P.longwangshana* and *P.gutianshana* were deposited in the Entomological Museum of China Agricultural University, Beijing, China (CAU). Photographs were captured by a Canon EOS 90D digital camera through a macro lens. Genitalia were prepared by boiling the apical portion of the abdomen in lactic acid for 0.5–1 h and then were examined and illustrations prepared by using a ZEISS Stemi 2000-C stereomicroscope. After examination, the removed abdomen was transferred to fresh glycerine and stored in a microvial on the pin with the specimen.

The present key is an emendation of the identification key of Kang et al. (2019) based on their comprehensive morphology data. Morphological terminology is based primarily on McAlpine (1981) and Fasbender (2014). The following abbreviations in figures are used: aea = ejaculatory apodeme, aes = aedeagal sclerite, alp = lateral ejaculatory process, as = sperm sac, asa = subapical sclerite of aedeagus, epand = epandrium, gas = apical stylus of gonostylus, gbl = basal lobe of gonostylus, goncx = gonocoxite, gonst = gonostylus, hypd = hypandrium, pm = paramere, prct = proctiger, sur = surstylus.

## Taxonomy

### Key to Chinese species of *Ptychoptera*

**Table:** 

1	Wing with r-m beyond fork of Rs, Rs not longer than r-m (Fig. 2c)	**2**
–	Wing with r-m before or at fork of Rs, Rs at least 1.5 times length of r-m (Figs 4e, 5a, b)	**8**
2	Mesopleuron mostly brown, epandrium yellow with caudal 1/2 brown	***P.circinans* Kang, Xue & Zhang, 2019 (Fujian)**
–	Mesopleuron uniformly yellow, epandrium uniformly yellow (Fig. 2a)	**3**
3	Gonostylus long and slender, about 1.5 times length of gonocoxite	***P.bannaensis* Kang, Yao & Yang, 2013 (Yunnan)**
–	Gonostylus short, as long as gonocoxite (Figs 3c, 5e)	**4**
4	Wing with a distinct spot at fork of R_4+5_, two spots at forks of R_1+2_ and M_1+2_ weak and nearly invisible	***P.lii* Kang, Yao & Yang, 2013 (Guizhou)**
–	Wing with three distinct spots at forks of R_1+2_, R_4+5_ and M_1+2_ separated (Fig. 2c) or forming a band (Figs 4e, 5a, b)	**5**
5	Basal 1/3 of second tergum of abdomen yellow with a median brown spot; lobe on middle area of gonostylus digitiform, slender	***P.lushuiensis* Kang, Yao & Yang, 2013 (Yunnan)**
–	Basal 1/3 of second tergum of abdomen uniformly brown (Fig. 2a); lobe on middle area of gonostylus broad, tongue-shaped (Fig. 3c)	**6**
6	Abdomen with 5^th^ and 6^th^ terga dark brown (Fig. 2a), tip of gonostylus with a hook-shaped ventral lobe (Fig. 3c)	**7**
–	Abdomen with 5^th^ and 6^th^ terga mostly yellow, tip of gonostylus with a digitiform ventral lobe (Nakamura and Saigusa 2009)	***P.formosensis* Alexander, 1924 (Taiwan; Japan)**
7	Sixth and 7^th^ sterna yellow, tip of surstylus curved up when viewed from the lateral side (Fig. 3a), retrose basal projection on inner side with tip bilobate (Fig. 3c), paramere with a pair of hook-shaped projections and a pair of conical projections (Fig. 3c), subapical sclerite of aedeagus serrated with five teeth (Fig. 3f)	***P.tianmushana* Shao & Kang, sp. nov. (Zhejiang)**
–	Sixth and 7^th^ sterna mostly brown, tip of surstylus not curved up when viewed from the lateral side, retrose basal projection on inner side not bilobate at tip, paramere with a pair of slender L-shaped projections, subapical sclerite of aedeagus serrated with two teeth	***P.emeica* Kang, Xue & Zhang, 2019 (Sichuan)**
8	Mesopleuron yellow (Fig. 2a)	**9**
–	Mesopleuron black (Fig. 4a–c)	**12**
9	Wing with bands and marks (Figs 2c, 4e, 5a, b)	**10**
–	Wing without band or mark	**11**
10	Base of Rs with an elliptic mark, abdomen with sterna yellow	***P.qinggouensis* Kang, Yao & Yang, 2013 (Neimenggu)**
–	Base of Rs without mark, abdomen with sterna black	***P.clitellaria* Alexander, 1935 (Sichuan)**
11	Wing with r-m its own length before fork of Rs, epandrium without long surstylus	***P.separata* Kang, Xue & Zhang, 2019 (Xizang)**
–	Wing with r-m close to fork of Rs, epandrium with a pair of long surstylus	***P.wangae* Kang, Yao & Yang, 2013 (Yunnan)**
12	Epandrium with surstylus curved downward (Figs 3a, 5c)	**13**
–	Epandrium with surstylus not curved downward	**14**
13	Wing with an elliptic mark at middle of CuA_1_ (Fig. 5b), tip of surstylus bifurcated (Fig. 5c)	***P.gutianshana* Yang & Chen, 1995 (Zhejiang)**
–	Wing without mark at middle of CuA_1_ (Fig. 5a), tip of surstylus not bifurcated	***P.longwangshana* Yang & Chen, 1998 (Zhejiang)**
14	Gonostylus much longer than gonocoxite	***P.xinglongshana* Yang, 1996 (Gansu)**
–	Gonostylus not longer than gonocoxite (Figs 3c, 5e)	**15**
15	Wing with an elliptic mark at middle of CuA_1_ (Fig. 4a, b, e)	***P.bellula* Alexander, 1937 (Jiangxi, Zhejiang)**
–	Wing without mark at middle of CuA_1_ (Figs 2c, 5a)	**16**
16	Abdomen with 2^nd^ and 3^rd^ terga brownish black; surstylus digitiform and broad basally, curved inwards at middle	***P.lucida* Kang, Xue & Zhang, 2019 (Xinjiang)**
–	Abdomen with 2^nd^ and 3^rd^ terga mostly yellow; surstylus flat and acinaciform, middle of inner edge slightly swollen (Kang et al. 2019)	***P.yankovskiana* Alexander, 1945 (Neimenggu; Korea)**

#### Ptychoptera tianmushana

Taxon classificationAnimaliaDipteraPtychopteridae

Shao & Kang sp. nov.

C04FF001-C9AE-5F2E-A544-FDF4429C6B56

http://zoobank.org/85E310AB-B6CC-4C07-841A-4E2BED7ED0C5

Figures 2 , 3

##### Type material.

***Holotype*** male (QAU), China, Zhejiang Province, Lin’an District, Mount Tianmushan, 2019.V.15, Xiao Zhang.

##### Diagnosis.

Wing marked with two brown bands. Epandrium bilobed, each lobe strongly elongated and forming a long surstylus, surstylus broadest at base, tapering and curved downward distally to middle, curving up at tip. Gonostylus with four projections and lobes. Hypandrium rectangular, posterior margin with a V-shaped projection bearing dense long hairs posteriorly, a triangular projection bearing dense short hairs laterally and a pair of elliptic projections bearing dense long hairs posteriorly.

##### Description.

**Male.** Body length 7.0 mm, wing length 8.0 mm.

Head mostly dark brown, except gena yellow with a black elliptical spot intermediately; clypeus yellow; hairs on head dark brown. Compound eyes black without pubescence. Antenna with scape, pedicel and basal half of 1^st^ flagellomere yellow, other flagellomeres light brown, hairs on antenna brown. Proboscis yellow with brown hairs. Palpus yellow with last segment light brown, hairs brown.

**Figure 2.**
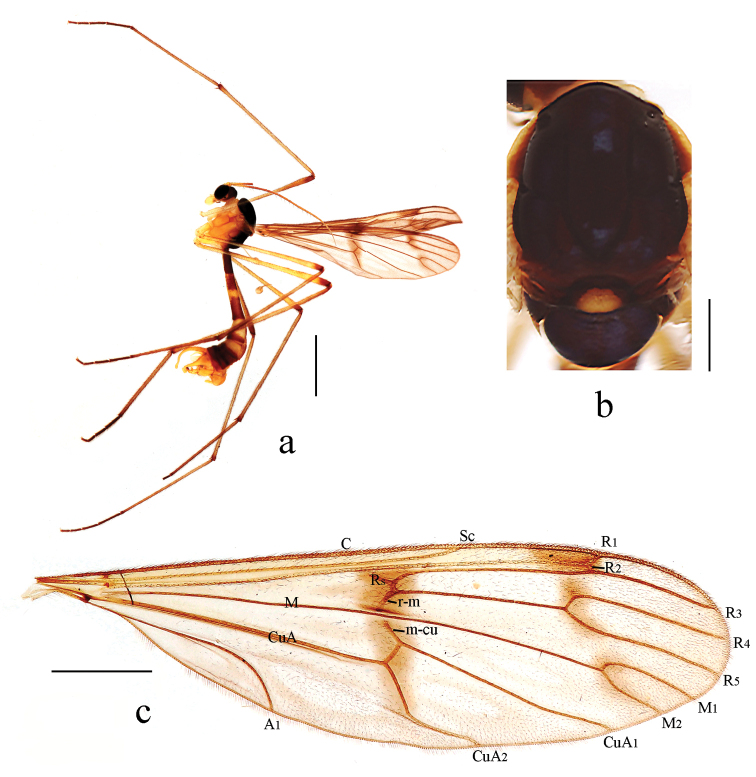
*Ptychopteratianmushana* sp. nov. **a** habitus of male, lateral view **b** thorax, dorsal view **c** wing. Scale bars: 2.0 mm (**a**); 0.5 mm (**b**); 1.0 mm (**c**).

Thorax (Fig. 2b). Pronotum yellow. Propleuron yellow. Mesonotum mostly dark brown with middle area of scutellum yellow and lower half of laterotergite yellow. Mesopleuron uniformly yellow. Coxae and trochanters yellow; fore femur yellow and gradually darkened apically; mid and hind femora yellow with brown ring apically; tibiae yellow with brown ring apically; 1^st^ tarsomere yellow brown and gradually darkened apically, 2^nd^ to 5^th^ tarsomeres uniformly dark brown. Hairs on legs brown. Relative length of 1^st^ to 5^th^ tarsomeres in hind leg as 11.2 : 2.8 : 1.6 : 1 : 1. Wing (Fig. 2c) 3.4 times as long as wide, subhyaline, marked with two brown bands as follows: median band broad and distinct, extending from basal part of cell r_2+3_ to fork of CuA; subapical band extending from anterior margin of wing, covering tip of R_1_ and R_2_, fork of R_4+5_, and extending to fork of M_1+2_, slightly separated into three marks. Veins brown; Sc ending in C not at level of basal third of R_2+3_; Rs straight, as long as r-m. Haltere and prehaltere pale yellow with brown hairs.

Abdomen. First tergum brown, 2^nd^ tergum brown with middle quarter area yellow, 3^rd^ and 4^th^ terga pale yellow with caudal third brown, 5^th^ to 7^th^ terga uniformly brown. Sterna pale yellow. Hairs on abdomen brown.

**Figure 3.**
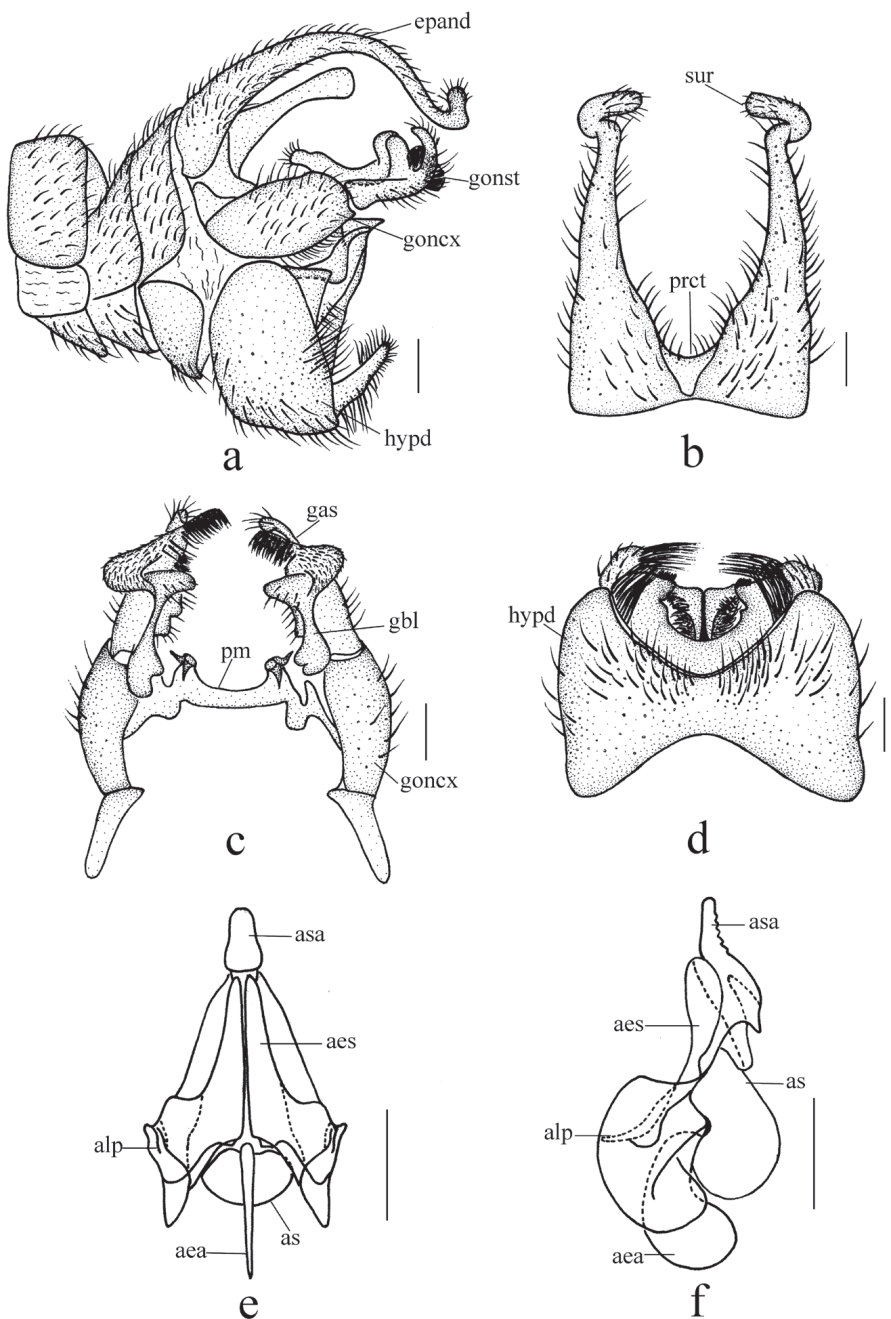
*Ptychopteratianmushana* sp. nov. **a** male genitalia, lateral view **b** epandrium, dorsal view **c** gonocoxite and gonostylus, dorsal view **d** hypandrium, ventral view **e** aedeagus, anterior view **f** aedeagus, lateral view. Scale bars: 0.2 mm.

Male genitalia yellow. Epandrium (Fig. 3b) bilobed, each lobe strongly elongated and forming a long surstylus, surstylus broadest at base, tapering and curved downward distally to middle, curving up at tip, with brown hairs; proctiger with short hairs. Gonocoxite (Fig. 3c) long and stout, 3 times as long as wide, basal apodeme two-thirds as long as gonocoxite; paramere with a pair of hook-shaped projections and a pair of conical projections. Gonostylus (Fig. 3c) with four projections and lobes: a strong retrose basal projection on inner side with tip bilobate; a broad tongue-shaped lobe on middle area with several long hairs apically; a dumbbell-shaped apical lobe broad basally with uniformly short hairs, apical with dense short setae; an apical hooked projection with several long hairs. Hypandrium (Fig. 3d) rectangular, anterior margin concaved medially, posterior margin with a V-shaped projection bearing dense long hairs posteriorly, a triangular projection bearing dense short hairs laterally and a pair of elliptic projections bearing dense long hairs posteriorly. Aedeagus (Fig. 3e, f): subapical sclerite tongue-shaped, slightly caved bilaterally, apex of subapical sclerite round; subapical sclerite serrated with five teeth when viewed from lateral side.

**Female.** Unknown.

##### Distribution.

China (Zhejiang).

##### Etymology.

The species is named after the type locality Mount Tianmushan.

##### Remarks.

This new species is very similar to *P.emeica* from China but can be separated from it by the 6^th^ and 7^th^ sterna of abdomen being yellow, the tip of the surstylus being curved up when viewed from the lateral side, the tip of the retrose basal projection on inner side being bilobate, the paramere having a pair of hook-shaped projections and a pair of conical projections, and the subapical sclerite of aedeagus being serrated with five teeth. In *P.emeica*, the 6^th^ and 7^th^ sterna of abdomen are mostly brown, the tip of the surstylus is not curved up when viewed from the lateral side, the tip of the retrose basal projection on inner side is not bilobate, the paramere have a pair of slender L-shaped projections, and the subapical sclerite of aedeagus is serrated with two teeth (Kang et al. 2019). In ecology, they live together with similar habitat. The adult of *P.tianmushana* was collected on the plants in close to streams and adults of *P.emeica* often found at the margins of streams or in wet forests. This new species is also similar to *P.formosensis* from China and Japan but can be separated from it by the 5^th^ and 6^th^ terga of abdomen being dark brown, the tip of the surstylus being curved up, the tip of the retrose basal projection on inner side being bilobate, the tip of gonostylus having a hook-shaped ventral lobe. In *P.formosensis*, the 5^th^ and 6^th^ terga of abdomen are mostly yellow, the tip of the surstylus is not curved up, the tip of the retrose basal projection on inner side is not bilobate, the tip of gonostylus have a digitiform ventral lobe (Alexander 1924; Nakamura and Saigusa 2009).

#### Ptychoptera bellula

Taxon classificationAnimaliaDipteraPtychopteridae

Alexander, 1937

85CD53CB-4BF2-5916-B5C4-F2C1D2CA7D0C

Figure 4

Ptychoptera bellula Alexander, 1937: 367. Type locality: Hong San, Jiangxi (China).

##### Specimens examined.

***Paratypes*:** 2 females (USNM), China: Jiangxi Province, Xunwu District, Hong San (1000–1053 m), 1936.VI.27–28, Judson Linsley Gressitt. **Other material**: 1 female (USNM), China: Zhejiang Province, Deqing District, Mount Moganshan, 1936.V.29, Père Octave Piel.

##### Diagnosis.

General coloration black. Haltere black with base of stem yellow. Wing with heavily brown bands and marks as follows: a large triangular brown mark in base of cells R and M; a irregular quadrate brown mark at origin of Rs; an elliptic brown mark at midlength of CuA_1_; a small oval brown mark at tip of A; median band extending from R to the bend in distal section of CuA_2_; subapical band extending from anterior margin of wing, covering tip of R_1_ and R_2_, to tip of M_2_; Rs about three-fifths the length of R_4+5_, 4 times the length of r-m.

##### Distribution (new record in bold).

China (Jiangxi, **Zhejiang**).

##### Remarks.

The determined specimen of this species collected from Zhejiang was identified by Alexander in 1939, but was not officially published to record. We re-determined it during the study and now record this species from Zhejiang for the first time. For descriptions and illustrations of this species, see Alexander (1937) and Krzeminski and Zwick (1993).

**Figure 4.**
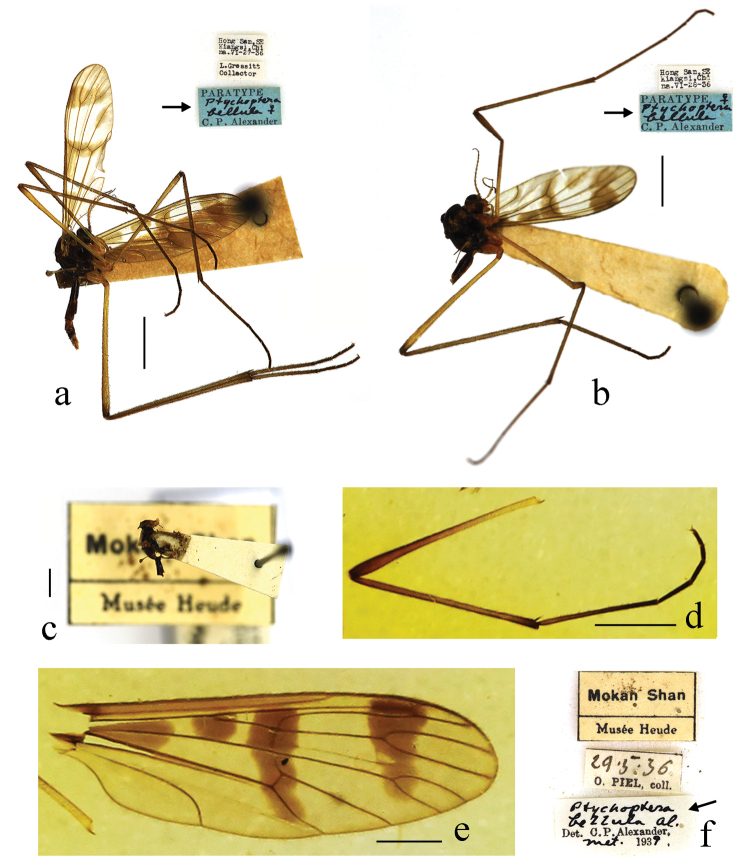
*Ptychopterabellula* Alexander, 1937 **a** habitus of one paratype, lateral view **b** habitus of the other paratype, lateral view **c–f** one female identified by Alexander (**c** habitus of female, lateral view **d** one leg **e** wing **f** collection and identification labels). Scale bars: 2.0 mm (**a–d**); 1.0 mm (**e**).

#### Ptychoptera longwangshana

Taxon classificationAnimaliaDipteraPtychopteridae

Yang & Chen, 1998

0728CE90-1AD6-5B54-9A1E-764099AB5C0E

Figure 5a

Ptychoptera longwangshana Yang & Chen, 1998: 240. Type locality: Mount Longwangshan, Anji, Zhejiang (China).

##### Specimens examined.

***Holotype*** male (CAU), China: Zhejiang Province, Anji District, Mount Longwangshan, 1996.VI.12, Chikun Yang.

##### Diagnosis.

Thorax mostly black. Wing marked with two brown marks and two brown bands. Surstylus of epandrium tapering and curved downward distally, curving up at tip. Gonocoxite wide and gonostylus short and small. Apical part of hypandrium with a pair of curved up and trough-shaped projections.

##### Description.

**Figure 5.**
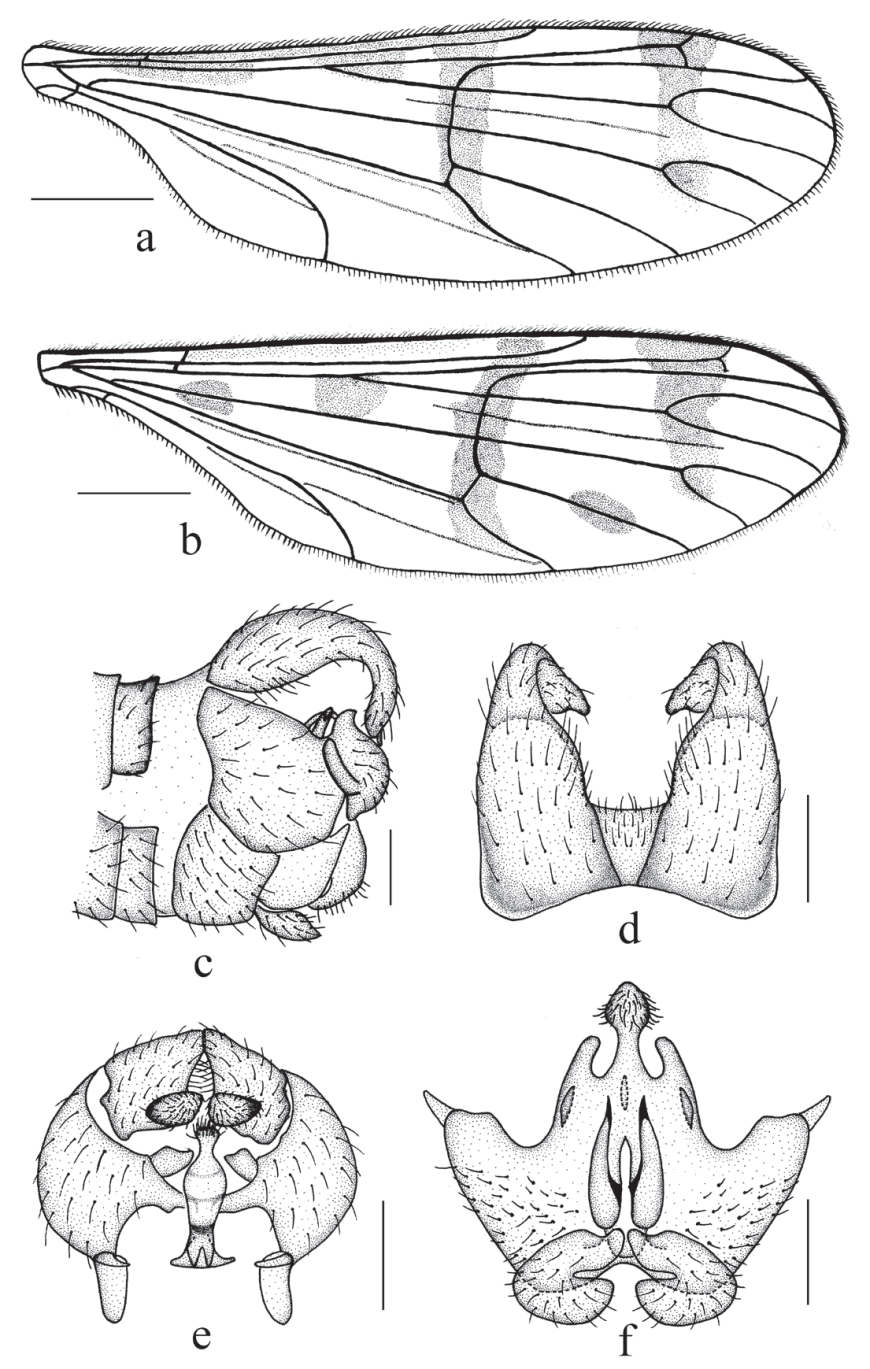
*Ptychopteralongwangshana* Yang & Chen, 1998 and *Ptychopteragutianshana* Yang & Chen, 1995 **a** wing of *P.longwangshana***b–f***P.gutianshana* (**b** wing **c** male genitalia, lateral view **d** epandrium, dorsal view **e** gonocoxite and gonostylus, dorsal view **f** hypandrium, ventral view). Scale bars: 1.0 mm (**a, b**); 0.2 mm (**c–f**).

Wing length 8.0 mm. Wing (Fig. 5a) 3.2 times as long as wide, subhyaline, marked with two brown marks and two brown bands as follows: an oval brown mark at base of R, a triangular brown mark at base of Rs; median band extending from anterior margin of wing, covering base of R_2+3_ and r-m, to middle section of CuA; subapical band extending from anterior margin of wing, covering tip of R_1_ and R_2_, to M_1+2_ fork, slightly separated into two marks. Veins brown; Sc ending in C at level of basal third of R_2+3_; Rs straight, 3 times the length of r-m.

##### Distribution.

China (Zhejiang).

##### Remarks.

Only one wing of the holotype was available during the study, while the rest of the holotype was not found in CAU. For a description and illustration of this species, also see Yang and Chen (1998).

#### Ptychoptera gutianshana

Taxon classificationAnimaliaDipteraPtychopteridae

Yang & Chen, 1995

E0C7DE12-F576-5975-9AD5-B11A95D825DE

Figure 5b–f

Ptychoptera gutianshana Yang & Chen, 1995: 180. Type locality: Mount Gutianshan, Kaihua, Zhejiang (China).

##### Specimens examined.

***Holotype*** male (CAU), China: Zhejiang Province, Kaihua District, Mount Gutianshan (350 m), 1993.IV.15, Hong Wu (light trap).

##### Diagnosis.

Wing marked with three brown marks and two brown bands. Surstylus of epandrium broadest at base, tapering and curved downward distally, forked at tip. Paramere with a pair of flaky projections and a vase-shaped projection. Gonostylus irregular rectangular with an elliptic basal projection on inner side with short hairs. Hypandrium trapeziform, anterior margin with a pair of C-shaped projections, middle area with a pair of digitiform projections, posterior margin with a pair of digitiform projections and a papillary projection.

##### Description.

Wing length 7.5 mm. Wing (Fig. 5b) 3.6 times as long as wide, subhyaline, marked with three brown marks and two brown bands as follows: three elliptic brown marks at base of Rs, at base of M and at midlength of CuA_1_; median band extending from anterior margin of wing, covering base of R_2+3_ and r-m, to the bend in distal section of CuA_2_; subapical band extending from anterior margin of wing, covering tip of R_1_, R_2_ and fork of R_4+5_, to fork of M_1+2_. Veins brown; Sc ending in C at level of basal half of R_2+3_; Rs straight, 3 times the length of r-m.

Male genitalia black. Epandrium (Fig. 5d) bilobed, each lobe strongly elongated and forming a long surstylus, surstylus broadest at base, tapering and curved downward distally, forked at tip, with brown hairs; proctiger with short hairs. Gonocoxite (Fig. 5e) short and swollen, 2 times as long as wide, basal apodeme three-eighths as long as gonocoxite; paramere with a pairs of flaky projections and a vase-shaped projection. Gonostylus (Fig. 5e) irregular rectangular with an elliptic basal projection on inner side with dense short hairs. Hypandrium (Fig. 5f) trapeziform, anterior margin with a pair of C-shaped projections, middle area with a pair of digitiform projections, posterior margin with a pair of digitiform projections and a papillary projection, papillary projection with dense short hairs.

##### Distribution.

China (Zhejiang).

##### Remarks.

Only two wings and male genitalia of the holotype were available during the study, while the paratype and the rest of the holotype were not found in CAU. For description and illustration of this species, also see Yang and Chen (1995).

## Supplementary Material

XML Treatment for Ptychoptera tianmushana

XML Treatment for Ptychoptera bellula

XML Treatment for Ptychoptera longwangshana

XML Treatment for Ptychoptera gutianshana
